# Social Media Insights During the COVID-19 Pandemic: Infodemiology Study Using Big Data

**DOI:** 10.2196/27116

**Published:** 2021-07-16

**Authors:** Huyen Thi Thanh Tran, Shih-Hao Lu, Ha Thi Thu Tran, Bien Van Nguyen

**Affiliations:** 1 National Taiwan University of Science and Technology Taipei Taiwan; 2 Ho Chi Minh City University of Law Ho Chi Minh Vietnam; 3 Dynimlabs Oulu Finland

**Keywords:** COVID-19, Vietnam, public attention, social media, infodemic, issue-attention cycle, media framing, big data, health crisis management, insight, infodemiology, infoveillance, social listening

## Abstract

**Background:**

The COVID-19 pandemic is still undergoing complicated developments in Vietnam and around the world. There is a lot of information about the COVID-19 pandemic, especially on the internet where people can create and share information quickly. This can lead to an infodemic, which is a challenge every government might face in the fight against pandemics.

**Objective:**

This study aims to understand public attention toward the pandemic (from December 2019 to November 2020) through 7 types of sources: Facebook, Instagram, YouTube, blogs, news sites, forums, and e-commerce sites.

**Methods:**

We collected and analyzed nearly 38 million pieces of text data from the aforementioned sources via SocialHeat, a social listening (infoveillance) platform developed by YouNet Group. We described not only public attention volume trends, discussion sentiments, top sources, top posts that gained the most public attention, and hot keyword frequency but also hot keywords’ co-occurrence as visualized by the VOSviewer software tool.

**Results:**

In this study, we reached four main conclusions. First, based on changing discussion trends regarding the COVID-19 subject, 7 periods were identified based on events that can be aggregated into two pandemic waves in Vietnam. Second, community pages on Facebook were the source of the most engagement from the public. However, the sources with the highest average interaction efficiency per article were government sources. Third, people’s attitudes when discussing the pandemic have changed from negative to positive emotions. Fourth, the type of content that attracts the most interactions from people varies from time to time. Besides that, the issue-attention cycle theory occurred not only once but four times during the COVID-19 pandemic in Vietnam.

**Conclusions:**

Our study shows that online resources can help the government quickly identify public attention to public health messages during times of crisis. We also determined the hot spots that most interested the public and public attention communication patterns, which can help the government get practical information to make more effective policy reactions to help prevent the spread of the pandemic.

## Introduction

### Background

The COVID-19 pandemic situation remains complicated, with nearly 82 million infection cases worldwide as of January 1, 2021 [1]. Due to Vietnam’s shared 1350 km land border with China, it was considered at high risk of an uncontrollable outbreak [2]. Yet Vietnam learned many lessons from the severe acute respiratory syndrome (SARS) epidemic when it failed to properly assess the infection risk from patients coming from the epidemic area for treatment at the Vietnamese French hospital, which triggered the SARS outbreak within its borders. Ultimately, Vietnam was the first country that the World Health Organization (WHO) removed from its list of those with community SARS infections [3]. During the first wave of the COVID-19 pandemic in Vietnam, the government quickly evaluated the novel coronavirus as a *strange and dangerous* virus with a high transmission risk that could easily result in an outbreak. The Vietnamese government executed preventive measures early, taking action a month before the WHO declared a Public Health Emergency of International Concern. The outcomes were impressive, as only 415 infections and no deaths were reported between January and June 2020 [4,5]. Following more than 3 consecutive months without cases of community spread infection, the second wave of the COVID-19 outbreak in Vietnam began when the 416th patient was declared infected in Danang on July 25, 2020. Vietnam recorded its first COVID-19 deaths during this period [6].

Pandemics are inherently negative situations; therefore, COVID-19–related news usually includes negative information such as infection rates, deaths, and quarantine information. Being surrounded by negative information can increase negative emotions, thereby driving perceptions of pandemic-related risk [7,8]. Unlike the SARS epidemic in 2003, connecting with potential medical users still mainly relies on email and personal communication (rather than other internet tools) to connect with each other and share information [9]. Many people have actively used the internet as their main source of information about the COVID-19 pandemic. However, the substantial amount of information in cyberspace may confound internet users who are trying to find and correctly evaluate reliable sources. This potentially harmful situation is known as an “infodemic” [10], which the WHO [11] defines as “an overabundance of information—some accurate and some not—that makes it hard for people to find trustworthy sources and reliable guidance when they need it” [12]. Therefore, understanding the dynamics of public attention during a pandemic such as COVID-19 is necessary to help governments, health ministries, or health educators design better guidelines to promote disease prevention and self-protection to return to social life and the “new normal” after resolution of the COVID-19 pandemic [13]. Previous research related to this field focused on analyzing community attention on a specific social media platform that is popular in the researchers’ country or region. Abd-Alrazaq et al [14] discussed Twitter users’ top concerns during the COVID-19 pandemic by analyzing collected data in English. Ahmad and Murad [15] conducted an online survey on Facebook to determine how social media has affected people during the COVID-19 pandemic. Another study [16] examined hot search lists on Sina Microblog, China’s most popular social media platform, to learn about public attention to COVID-19 in China. Several research papers have focused on public reaction to the COVID-19 pandemic in Vietnam. Trevisan et al [17] examined the country’s reaction to and control of the pandemic; another study [18] described the pattern of the pandemic’s early stage in Vietnam using a secondary data set provided by the country’s Ministry of Health. Other researchers [19] used a survey to understand COVID-19 risk perception from socioeconomic and media attention perspectives.

In this study, we analyze big data collected from popular online sources where people obtain, create, or discuss news and information in Vietnam, including Facebook, news websites, YouTube, forums, blogs, Instagram, and e-commerce sites. Data were collected from December 2019 to November 2020 to offer a wider view from diverse sources and a longer observation period. We analyzed this data to describe a pattern of the social reaction during two different waves of the COVID-19 pandemic in Vietnam using the issue-attention cycle and media framing theories as foundations to develop our research questions.

### Issue-Attention Cycle Theory

The risk of COVID-19 infection is still high worldwide given that vaccination is not yet widely used and some countries are trying to resume normal commercial operations, including commercial flights, in an attempt to recover economically from the consequences of the pandemic. The need to seek and discuss information during a pandemic crisis like COVID-19 is obvious. However, many people try to simplify complex information or rely on their current beliefs; this may create conflict if they must force new information into previous constructs. Facing the risk of illness or death, as in the COVID-19 pandemic, can change people’s attitudes toward “accepting information, handling and taking action on it” [20-22]. In 1972, Downs [23] introduced the issue-attention cycle theory that refers to the attention trend line an environmental issue could receive from the public or media, as described in five main stages. In the first stage, only experts or a small number of people interested in the issue are aware of it. In the second stage, the issue captures more attention as awareness of it increases; at this stage, people are optimistic that the problems will be solved one way or another. The third stage is marked by chaos, which peaks when people realize the issues might be far different from their expectations, out of their control, and present with high financial or social benefit costs. A steady drop in public attention to the issue characterizes the fourth stage, which is known as the postproblem phrase. The final stage is marked by replacement of the concerning issues in public attention [23].

However, some researchers argued that the issue-attention cycle can differ depending upon culture [24] and in cases of epidemic hazards [25]. Moreover, the issue-attention cycle is not always fully integrated or fully explanatory in some health-related research, as evidenced by the “Charlie Sheen effect” phenomenon, introduced by Ayers et al [26] in 2016 when they used results from Google’s search engine data set to show the correlation between actor Charlie Sheen’s disclosure of his HIV-positive status with the level of public attention to HIV and its prevention.

Our study investigates public attention during the COVID-19 pandemic by examining internet discussion volume to find patterns and determine similarities or differences to the issue-attention cycle theory. The amount of public discussion on social media has changed over time based on the public’s response to each real event that occurred during the pandemic. Capturing the amount of public discussion not only helps to point out or compare patterns in issue-attention cycle theory but also shows how the public’s attention to specific events is different. From there, it is possible to help the government and stakeholders evaluate the severity of each event to the public and from there learn lessons for possible pandemic prevention in the future. Hence, the research questions related to this theory are:

- RQ1: What is the level (volume) of public attention to COVID-19 in this study?
- RQ2: What does the pattern of public attention to the pandemic look like?

Throughout the COVID-19 pandemic, a concurrent infodemic has bombarded the public, hindering the reception of reliable information sources so citizens can follow recommendations and protect themselves. Therefore, in addition to pointing out patterns of pandemic-related discussions, it is necessary to dig deep into sources that get the most public attention, which can help government and disease control centers stop inaccurate news that has reached a large number of people in a timely manner. These patterns can also help identify popular public channels to help legitimate agencies broadcast disease prevention messages more efficiently. Additionally, analyzing the public sentiment about the pandemic can help governments and the Centers for Disease Control and Prevention deliver more accurate prevention messages to appease public anxiety and insecurity. Therefore, we developed the third and fourth research questions:

- RQ3: Which types of sources gained the most public attention and engagement during the pandemic?
- RQ4: How did people react to the pandemic, as measured by expression of their emotions on social media?

### Media Framing Theory

The mechanism by which individuals create a clear conceptualization or reorient their thoughts about an issue is referred to as framing theory. The concept is based on the acceptance of an issue that can be presented from a number of viewpoints and is perceived as having implications for different principles or factors [27]. Frames matter, especially in communications meant to influence an audience’s attitudes and behaviors. Frame use is learned and may be adopted from person to person. Previous studies have shown that politicians have been inspired by the communication styles of other politicians, the media, or even citizens [28-30]. It is understandable that even in conversation and discussion with others, individuals typically adopt the frames that they have learned [30-32].

The explosive growth of information technology and social networking in the digital age has resulted in changes to the concept of “news,” which was once considered the product of a journalist [33]. The concept has broadened now that anyone can create news by uploading it to the internet in the form of pictures, text, video, etc [34]. Sometimes this news is only 140 characters long [35], and its credibility depends on the number of interactions garnered from readers, including likes, shares, and comments [36]. The nature of this news formulation and discourse is dynamic, so it is important to examine how frames used to report on epidemic hazards may change and develop over time [31,37,38]. Understanding the critical role framing plays in communication, scholars have monitored frames over the past decade to detect patterns in problem descriptions, analyze media attention, and investigate differences across forms of media [39]. Thus, in our study, we seek answers to the following questions:

- RQ5: What frames are used and how frequently are they used in communications that occur during the pandemic? What main topics gained the most discussion and attention during the COVID-19 pandemic?
- RQ6: Were different types of frames used during the first and second waves of the COVID-19 pandemic in Vietnam?

## Methods

All information related to COVID-19 in Vietnam was obtained from the Ministry of Health of Vietnam’s official COVID-19 disease page [6] and the website thuvienphapluat.vn [40], an electronic library of legal documents issued by the Vietnamese government. We used this information to create a foundation for collecting data from social platforms and as a basis for comparison with the results obtained after data analysis.

### Data Collection and Processing

This study aims to understand the public reaction to the COVID-19 pandemic via discussions among Vietnamese people on social media. We used SocialHeat, a fee-based social listening tool developed and sponsored by YouNet Group, to crawl data while following the terms of use from 7 types of sources: Facebook, Instagram, news, blogs, forums, e-commerce sites, and YouTube*.* SocialHeat collected public data on social networks in real time using COVID-19–related keywords (coronavirus, nCoV, SARS-CoV-2, COVID-19, Covid). Counted topics were written in Vietnamese only and pulled from the Facebook application programming interface (API), Instagram API, YouTube API, and Google API (for news, blogs, e-commerce, and forum websites). All data from spam and noise mentions were deleted by applying deep learning and natural language processing in the SocialHeat system (the data set still might contain seeding posts or brand commercial posts, but the numbers of those posts are negligible).

The data set was collected from December 1, 2019, to November 13, 2020, from 63 million Facebook IDs (pages, individual profiles, and groups), 1.2 million YouTube accounts, 9000 news websites, and 300 forums in Vietnam. On account of the amount of data and technology limitations, we divided the timeline into 7 periods to crawl data, then reconnected the data in a complete and continuous timeline. To divide the timeline, we relied on highlighted events that took place during the period observed (December 1, 2019, to November 13, 2020). Specifically, we used data tracking new daily infections in Vietnam, which was updated by the Ministry of Health of Vietnam [6] and included the four main stages of the COVID-19 outbreak in Vietnam as described by La et al [41] (as of April 4, 2020) to inform additional development into 7 main phases (Table 1).

**Table 1:** The 7 periods of the COVID-19 pandemic in Vietnam.

Period	Date	Total days, n	Events	
1	Before January 23, 2020	54	No confirmed cases in Vietnam	
2	January 23 to February 26, 2020	35	First confirmed case in Vietnam; 16th infected case discharged from hospital	
3	February 27 to March 5, 2020	8	No new cases in Vietnam	
4	March 6 to March 31, 2020	26	17th infected case confirmed and more reported afterward	
5	April 1 to April 15, 2020	15	Implementation of social isolation	
6	April 16 to July 24, 2020	100	No new cases in the community	
7	July 25 to November 13, 2020	112	A new case in the community and the first deaths	

### Mention Trend Line

The volume of total mentions (a mention can be an original post, a comment, or a share) about COVID-19–related topics on digital channels, including Facebook, Instagram, news sites, forums, blogs, etc, was tallied and expressed by day to show how Vietnamese citizens reacted to COVID-19 pandemic–related events timeline-by-timeline. This study also integrates the real flow of facts and disease coping measures adopted by the government to analyze the relationship between government policies and peoples’ reactions during the pandemic.

### The 500 Most Engaging Sources

To explore which sources attracted the most attention and engagement, we calculated the total interactions on COVID-19–related topics across all ID sources (Facebook, YouTube, and Instagram) and unique links on news, blog, forum, and e-commerce sites, then ranked them in order from highest to lowest. The total interaction with an engaging source equal to the total COVID-19–related posts was posted by observed source, plus total likes, shares, and comments that those posts gained.

Facebook is the most popular social platform in Vietnam [42]. It is not only popular with individuals but also used for official brand fan pages, key opinion leaders (KOLs), TV channels, news, and government departments that use Facebook as a connecting bridge with customers, readers, citizens, etc. Therefore, we categorized Facebook accounts into 8 clusters: community pages, news, TV channels, KOLs, forums, groups, government, and unknown (minor accounts that could not be categorized into any source). Due to the limitations of hand categorization, we chose only the top 500 sources by mentions each period and categorized them for analysis.

### Top 50 Posts by Mentions

We analyzed top posts created during the COVID-19 pandemic to understand which topics attracted the most citizen attention and their associated reactions via discussion sentiment analysis. Top posts were COVID-19–related posts that gained the most mentions (shares, comments) on Facebook, Instagram, YouTube, news sites, blogs, e-commerce sites, and forums.

Previous studies about the information shared on social media by users during crisis events had different ways of classifying content based on real events. For example, Vieweg [43], who studies communications and behavior during mass emergencies, categorized the types of information that users create into three main groups: social environments (eg, caution, advice, medical attention, and offering help), built environment (eg, infrastructure damage), and physical environment (eg, weather forecast and general information about hazards). Based on research of Vieweg [43], Imran et al [44] has inherited and continues to categorize the content collected from researching on social media messages related to disasters into types of content such as caution and advice; casualties and damage; donations of money, goods, or services; people missing, found, or seen; and information source. Meanwhile, Mirbabaie et al [45] studied what happened on social media during the Hurricane Harvey incident to find lessons in dealing with the COVID-19 pandemic and classified the information into seven categories based on the information gathered during the data analysis process: official statement, news and crisis information, personal opinion, personal experience, forwarding message, solicitousness, and humor.

The research on the nature of information spread about the COVID-19 pandemic on Weibo by Li et al [46] classified content into 7 groups based on the previous work of Rudra et al [47] and Vieweg [43], including notifications or measures taken; donating money, goods, or services; emotional support; help seeking; doubt casting and criticizing; counter rumors; and policy reaction. In the process of applying the aforementioned classifications, we identified 5 types of content that appeared frequently but are not suitable for distribution into the 7 existing content groups, including caution and advice; international situation updates; medical issues, treatment, and vaccine; effects of the pandemic on the economy; and entertainment. Thus, in this study, the 50 posts with the most public engagement (interaction) were categorized and sorted into 12 groups of content.

### Sentiment Trend Line, COVID-19–Related Topics’ Keyword Frequency, and Social Networks

For all COVID-19 data downloaded from Facebook, Instagram, news sites, forums, blogs, etc, the SocialHeat tool excluded noise, spam, and advertising posts before using natural language software developed by the YouNet Company for sentiment classification and to extract the top 50 keywords’ frequency for the 7 observed periods.

The most frequently mentioned keywords for each period were analyzed and visualized using VOSviewer (Nees Jan van Eck and Ludo Waltman) [48]. A social network and clusters for each period were created using the keywords matrix, in which every two keywords are linked by co-occurrence frequency. In other words, the frequency of occurrence of two keywords in the same article will be shown through the link between two dots. The larger the dot, the more often the keyword appears. The thicker and closer the link between two dots (two keywords), the more frequency the two keywords will appear together.

## Results

### Total Discussions About COVID-19 on Social Media in Vietnam During the First Two Waves of the Pandemic

There was a total of 37,917,631 collectable mentions and 22,652,638 posts about COVID-19 from December 1, 2019, to November 13, 2020. *Collectable mentions* refers to mentions set in public mode on the online channels; therefore, only public data was collected due to privacy settings. The data set was summarized daily and put in chronological order (see Multimedia Appendix 1). Facebook was the channel that gained the most mentions (27,191,922 mentions, accounting for 96.4% of total mentions), while other channels shared the rest (forums: 232,131 mentions; news sites: 757,582 mentions; blogs: 1058 mentions; reviews: 224 mentions; e-commerce sites: 2231 mentions; YouTube: 20,599 mentions; Instagram: 1857 mentions).

There was a positive correlation between total collectable mentions on social media and daily new COVID-19 infection cases (β0=74,451.4; β1=9366.9; *P*<.001). In other words, the more new infection cases counted daily, the more posts and mentions of COVID-19 pandemic topics created on social platforms like Facebook, YouTube, Instagram, etc, and on websites including news sites, forums, blogs, etc.

Multimedia Appendix 1 indicates the Vietnamese public’s attention and reaction toward the two first waves of the COVID-19 pandemic. Data were divided into 7 periods (the same as those in Table 1) based on the highlighted events happening in Vietnam. During period 1 (December 1, 2019, to January 22, 2020) and especially before January 12, 2020, Vietnamese people paid little attention to information about the COVID-19 epidemic, although China recorded the first cases in Wuhan [49]. During period 2 (January 23, 2020, to February 26, 2020), public attention increased significantly when Vietnam confirmed that the first COVID-19 case in the country was from a Chinese traveler [50]. Period 3 (February 27, 2020, to March 5, 2020) saw very low public attention when no new infections were confirmed by the government. In period 4 (March 6, 2020, to March 31, 2020), total posts peaked with more than 1.2 million mentions about COVID-19 after the 17th case was confirmed. The highest total collectible mentions (1,255,175 mentions) were made on March 31, one day before the Vietnamese government’s implementation of a *social isolation* mandate throughout the country. Period 5 (April 1, 2020, to April 15, 2020) had a deep drop but a stable number of total and collectible mentions about COVID-19–related topics compared to period 4. Period 6 (April 16, 2020, to July 23, 2020) had a significant steady decrease in public attention toward the pandemic when the government removed the social isolation order and simultaneously did not report new community infection cases. However, there was a small fluctuation indicating increased discussions starting on June 22, 2020, and peaking July 1, 2020, with 320,089 discussions, due to information about a COVID-19 vaccine developed in Vietnam that was expected to be clinically tested in humans in October or November 2020. Additionally, a suspected COVID-19 infection had been discovered in Danang; public attention gradually decreased through period 7, when a community infection was confirmed in Danang.

### Sources With the Most Interaction During the First Wave of the COVID-19 Pandemic in Vietnam

#### Total Interactions on the Top 500 Most Engaging Sources

As shown in Table 2, the community page sources remained the most popular throughout the whole period. The remaining interactions were split among other types of sources, including news sites, KOLs, government sites, etc.

During period 1 (December 1, 2019, to January 22, 2020), news sources gained the most public reaction, and TV channel sources followed right after. After period 1, community page sources steadily earned the most engagement. This was especially true during period 2 (January 23, 2020, to February 26, 2020), period 4 (March 6, 2020, to March 31, 2020), period 5 (April 1, 2020, to April 15, 2020), and period 7 (July 25, 2020, to November 13, 2020), when around 50% of Vietnamese citizens’ interactions about the pandemic came from community page sources. Meanwhile, TV channel sources (periods 1, 2, 4, and 7) and KOL sources (periods 3, 5, and 6) alternated second place status in terms of engagement on COVID-19–related topics.

In contrast, forum (periods 2, 6, and 7) and government (periods 1 and 3) sources gained less total interaction.

**Table 2:** Total reactions on the top 500 most engaging sources.

Source	Period 1 (n=265,679), n (%)	Period 2 (n=3,217,036), n (%)	Period 3 (n=137,642), n (%)	Period 4 (n=39,200,553), n (%)	Period 5 (n=10,086,791), n (%)	Period 6 (n=567,114), n (%)	Period 7 (n=96,832,404), n (%)
Community page	63,251 (23.81)	1,600,342 (49.75)	50,549 (36.72)	21,392,949 (54.57)	4,734,704 (46.94)	170,131 (30.00)	42,131,669 (43.51)
Forum	5819 (2.19)	16,918 (0.53)	498 (0.36)	540,775 (1.38)	128,025 (1.27)	0 (0.00)	528 (0.00)
Government	346 (0.13)	85,434 (2.66)	0 (0.00)	1,350,699 (3.45)	431,201 (4.27)	98,005 (17.28)	8,340,435 (8.61)
Group	11,934 (4.49)	266,640 (8.29)	15,806 (11.48)	3,367,923 (8.59)	727,409 (7.21)	38,046 (6.71)	7,794,693 (8.05)
Key opinion leaders	39,234 (14.77)	309,160 (9.61)	45,529 (33.08)	3,889,108 (9.92)	1,903,164 (18.87)	165,804 (29.24)	11,732,842 (12.12)
News	73,982 (27.85)	346,683 (10.78)	15,043 (10.93)	3,300,849 (8.42)	724,832 (7.19)	63,080 (11.12)	10,681,121 (11.03)
TV channel	65,330 (24.59)	359,969 (11.19)	3863 (2.81)	4,107,847 (10.48)	1,384,393 (13.72)	26,499 (4.67)	16,057,180 (16.58)
Unknown	5783 (2.18)	231,890 (7.21)	6354 (4.62)	1,250,403 (3.19)	53,063 (0.53)	5549 (0.98)	93,936 (0.10)

#### The Average Interaction on the Top 500 Most Engaging Sources

Total interactions on the most engaging sources were calculated by summarizing the number of each source’s COVID-19–related posts, likes, shares, and comments. We analyzed the average interaction on the top 500 most engaging sources to understand the efficiency of each COVID-19–related post created by each source.

As can be seen from Table 3, government sources were leading in periods 2, 4, 5, and 6 with nearly 32% to 67% of the average interactions for the top 500 most engaging sources. News and TV channel sources alternated in the second position in periods 1 and 6 and periods 2, 3, 4, and 5 (TV channels). Period 7 was unique in that KOLs received the highest average engagement (2,330,684/3,745,249, 62.23%), followed by news from community sites (861,254/3,745,249, 23%).

**Table 3:** The average interaction on the top 500 most engaging sources.

Sources	Period 1 (n=10,167), n (%)	Period 2 (n=102,421), n (%)	Period 3 (n=3184), n (%)	Period 4 (n=1,111,044), n (%)	Period 5 (n=645,151), n (%)	Period 6 (n=12,802), n (%)	Period 7 (n=3,745,249), n (%)
Community page	427 (4.20)	5443 (5.31)	468 (14.70)	74,540 (6.71)	16,327 (2.53)	915 (7.15)	861,254 (23.00)
Forum	1940 (19.08)	5639 (5.51)	249 (7.82)	90,129 (8.11)	32,006 (4.96)	0 (0.00)	528 (0.01)
Government	346 (3.40)	42,717 (41.71)	0 (0.00)	450,233 (40.52)	431,201 (66.84)	4900 (38.28)	321,134 (8.57)
Group	385 (3.79)	5442 (5.31)	368 (11.56)	57,083 (5.14)	13,989 (2.17)	865 (6.76)	87,847 (2.35)
Key opinion leaders	162 (1.59)	3964 (3.87)	149 (4.68)	54,015 (4.86)	17,954 (2.78)	825 (6.44)	2,330,684 (62.23)
News	2000 (19.67)	11,556 (11.28)	1003 (31.50)	94,310 (8.49)	25,887 (4.01)	2426 (18.95)	67,245 (1.80)
TV channel	4666 (45.89)	17,998 (17.57)	644 (20.23)	228,214 (20.54)	81,435 (12.62)	2409 (18.82)	75,552 (2.02)
Unknown	241 (2.37)	9662 (9.43)	303 (9.52)	62,520 (5.63)	26,352 (4.08)	462 (3.61)	1005 (0.03)

### Top Posts About COVID-19 Topics With the Most Comments or Shares

The type of COVID-19–related content that received the most attention varied from time to time. Starting from phase 2 onward, the diversity of content types increased to include caution and advice, policy reaction, and international situation updates (Table 4).

**Table 4:** Top posts with the most comments or shares.

Categories	Period 1 (n=49,480), n (%)	Period 2 (n=337,865), n (%)	Period 3 (n=24,173), n (%)	Period 4 (n=1,375,260), n (%)	Period 5 (n=312,851), n (%)	Period 6 (n=986,403), n (%)	Period 7 (n=9,161,011), n (%)
Caution and advice	27,607 (55.79)	28,869 (8.54)	0 (0.00)	191,017 (13.89)	28,067 (8.97)	129,076 (13.09)	347,096 (3.79)
Notifications or measures have been taken	7116 (14.38)	13,222 (3.91)	2229 (9.22)	162,502 (11.82)	0 (0.00)	23,978 (2.43)	2,120,104 (23.14)
Donation money, goods, or services	0 (0.00)	0 (0.00)	0 (0.00)	12,167 (0.88)	4982 (1.59)	89,919 (9.12)	1,477,020 (16.12)
Emotional support	0 (0.00)	0 (0.00)	4905 (20.29)	328,056 (23.85)	153,652 (49.11)	195,632 (19.83)	970,817 (10.60)
Help seeking	0 (0.00)	0 (0.00)	0 (0.00)	9045 (0.66)	0 (0.00)	0 (0.00)	133,675 (1.46)
Doubt casting and criticizing	0 (0.00)	112,297 (33.24)	3284 (13.59)	186,581 (13.57)	2537 (0.81)	0 (0.00)	666,394 (7.27)
Counter rumors	0 (0.00)	55,397 (16.40)	0 (0.00)	0 (0.00)	0 (0.00)	0 (0.00)	0 (0.00)
Policy reaction	1226 (2.48)	116,869 (34.59)	11,642 (48.16)	289,200 (21.03)	112,505 (35.96)	0 (0.00)	1,132,238 (12.36)
International situation updating	13,531 (27.35)	11,211 (3.32)	1678 (6.94)	196,692 (14.30)	11,108 (3.55)	107,243 (10.87)	100,369 (1.10)
Medical issues: treatment, vaccine	0 (0.00)	0 (0.00)	0 (0.00)	0 (0.00)	0 (0.00)	432,389 (43.83)	0 (0.00)
Effects of the pandemic on the economy	0 (0.00)	0 (0.00)	435 (1.80)	0 (0.00)	0 (0.00)	8166 (0.83)	0 (0.00)
Entertainment	0 (0.00)	0 (0.00)	0 (0.00)	0 (0.00)	0 (0.00)	0 (0.00)	2,213,298 (24.16)

In period 1, when the first COVID-19 cases were found in Wuhan and had not yet spread to Vietnam, it is understandable that content concerning *caution and advice* received the most attention, accounting for 55.8% (27,607/49,480). Content about *international updates* was next, accounting for 27.3% (13,531/49,480). In period 2, when Vietnam confirmed that two Chinese tourists were infected with COVID-19 and that these were the first two cases of COVID-19 to appear in Vietnam, articles regarding *policy reaction* were of most interest, accounting for 34.6% (116,869/337,865); content about *doubt casting and criticizing* received almost equal attention, accounting for 33.2% (112,297/337,865). In the third stage when Vietnam had no community cases, people remained interested in topics classified as *policy reaction* (11,642/24,173, 48.2%) and began to pay attention to content on *emotional support* (4905/24,173, 20.3%). During phase 4, as community cases peaked and new infections were recorded, people were most interested in the topic of *emotional support* (328,056/1,375,260, 23.9%) and *policy reaction* (289,200/1,375,260, 21%). This was also the period when interest was shared between the greatest variety of content types with fairly similar distribution. In period 5, when the Vietnamese government applied a social isolation mandate, people were most concerned with *emotional support* (153,652/312,851, 49.17%) and *policy reaction* (112,505/312,851, 36.7%). During period 6, when Vietnam enjoyed 100 days of peace without news of community spread, articles on *medical issues* received the most attention (432,389/986,403, 43.87%) followed by *emotional support* (195,632/986,403, 19.87%). When community cases reappeared and though there were more COVID-19 deaths in Vietnam, peoples’ response was quite optimistic, with attention almost equally divided between the topics of *entertainment* (2,213,298/9,161,011, 24.3%) and *notifications or measures being taken* (2,120,104/9,161,011, 23.2%).

### Sentiment

**Figure 1:**
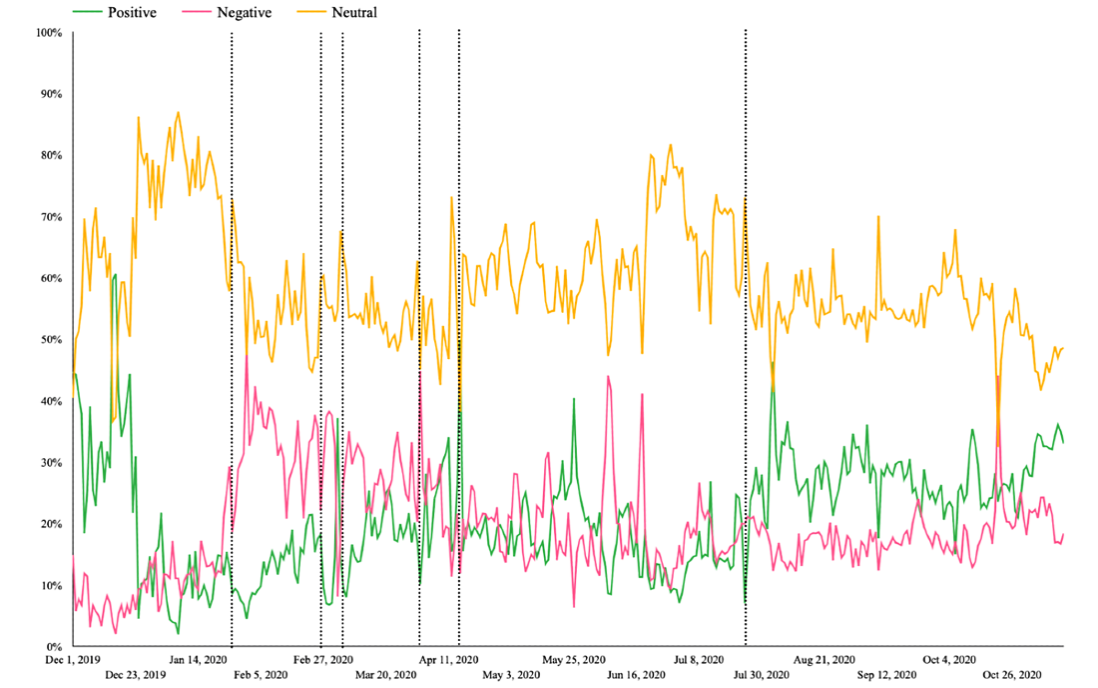
Sentiment trend line from December 1, 2019, to November 13, 2020.

After all text data related to COVID-19 was crawled, it was processed by a sentiment analysis tool developed by SocialHeat. All discussions were evaluated and sorted into one of three emotional categories, positive, negative, and neutral, based on natural language. In general, we found that people’s emotions when discussing COVID-19–related topics fluctuate and are unstable. Emotional neutrality almost always took first place. This is understandable because sources from government organizations and especially television and newspapers are expected to report “independent, reliable, accurate, and comprehensive information” [33]. However, in various time periods, negative and positive emotions alternated in second place. Positive emotions were expressed more often than negative emotions during the first and last periods. Negative emotions were expressed more than positive ones; most appear throughout stages of Vietnam’s first COVID-19 wave (periods 2, 3, 4, 5, and 6; see Figure 1).

During period 1, when Vietnam had not yet recorded any cases and the pandemic situation had just begun in China, people learned about COVID-19 through information from the Ministry of Health and the press, so their mentality was still stable and optimistic. In period 2, when the first cases were discovered in Vietnam, people become more confused and worried. In period 3, negative emotions exploded when patient 17 was confirmed and there was a risk of community disease spread. Anger, blame, and anxiety were evident through the negative emotions expressed in the text lines discussed on social networks at that time. In period 7 when Vietnam experienced its second wave of COVID-19 with the re-emergence of community infection and the first recorded COVID-19 deaths, the optimism shown through positive emotions overwhelms the negative emotions expressed during this period. People have gradually adapted to the pandemic after experiencing the first wave and have confidence in the government’s ability to control the pandemic; positive signals that a Vietnamese COVID-19 vaccine would soon enter the human testing phase may have also contributed to the positive outlook [51].

### Top Keywords’ Frequency and Social Network Analysis of Discussions on the Internet During the COVID-19 Pandemic in Vietnam

#### Top Keywords

The top 50 keywords were compiled and ranked in order from all discussions on the COVID-19 pandemic topic gathered during the study period. However, of the top 50, many keywords are synonyms, so we have grouped them into 36 keywords. The content of the top keywords was related to 4 main groups, including *COVID-19 pandemic and epidemic outbreaks* expressed through keywords such as epidemic, COVID-19, Vietnam, case, Danang, Hanoi, and Bach Mai hospital; *policy reactions* as shown through words including quarantine, against, prevention, mask, province, and government; *medical issues* expressed through patient, hospital, test, contact, infected, virus, and treatment; and *disease situation in the world* through words like situation, the United States, and money (see Table 5).

**Table 5:** Top 36 keywords for COVID-19–related topics during the COVID-19 pandemic in Vietnam from December 1, 2019, to November 13, 2020.

Rank	Word	Frequency, n
1	epidemic	16,373,688
2	COVID-19	10,720,319
3	patient	9,838,764
4	quarantine	9,783,246
5	medical	9,349,795
6	go	8,428,703
7	hospital	8,257,058
8	Vietnam	7,789,027
9	case	7,643,082
10	disease	6,397,632
11	Danang	5,621,518
12	against	5,208,937
13	city	5,066,441
14	infected	4,734,692
15	virus	4,099,245
16	situation	3,950,498
17	information	3,854,982
18	province	3,850,230
19	prevention	3,692,883
20	citizen	3,357,150
21	mask	3,316,868
22	way	3,310,041
23	test	2,784,690
24	contact	2,764,565
25	government	2,705,459
26	Hanoi	2,422,218
27	family	2,366,785
28	money	2,177,978
29	coronavirus	2,039,959
30	The US	1,903,865
31	vehicle	1,842,155
32	result	1,821,271
33	treatment	1,790,499
34	Bach Mai hospital	1,663,212
35	together	1,654,416
36	get sick	1,475,917

#### Social Network Co-occurrence of the 7 Periods

To better understand the context behind the most mentioned keywords and to highlight the top concerns about the COVID-19 pandemic expressed in internet discussions in each period in Vietnam, we extracted the top 50 keywords for each stage and visualized the associations between the keywords using VOSviewer software. The larger the dots, the more weight (frequency) that keyword possessed. The thicker and closer the link between two keywords, the more frequently both keywords appear.

**Figure 2:**
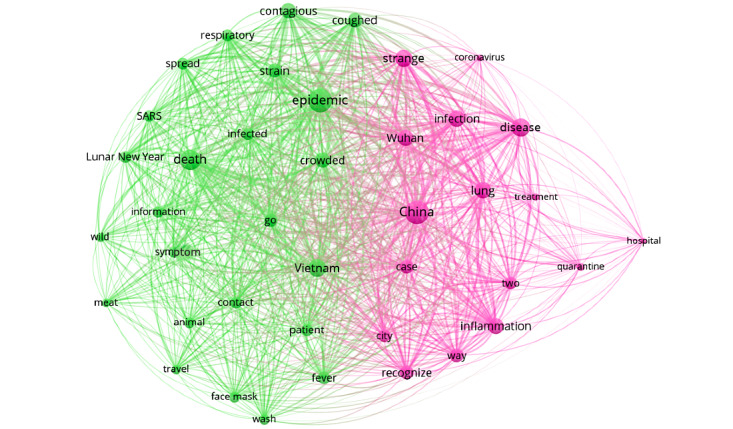
Co-occurrences of the top keywords in period 1. SARS: severe acute respiratory syndrome.

The relationship between the top keywords in period 1 when no infections were found in Vietnam is shown in Figure 2. The most prominent keywords were “China,” “epidemic,” “death,” “disease,” “strange,” “Wuhan,” and “inflammation.” The pink color cluster reflects the first awareness of COVID-19 infections in Wuhan, China at that time, as reflected by words including “China,” “Wuhan,” “strange,” “lung,” “coronavirus,” “quarantine,” etc. The green cluster represents the first information about COVID-19 that was communicated by the Vietnamese government to the people, and includes “epidemic,” “death,” “meat,” “wild,” “travel,” “respiratory,” etc, along with keywords that guide how to proactively prevent epidemics, especially during the Lunar New Year period, including “face mask,” “wash,” “go,” “travel,” “Lunar New Year,” “crowded,” etc.

**Figure 3:**
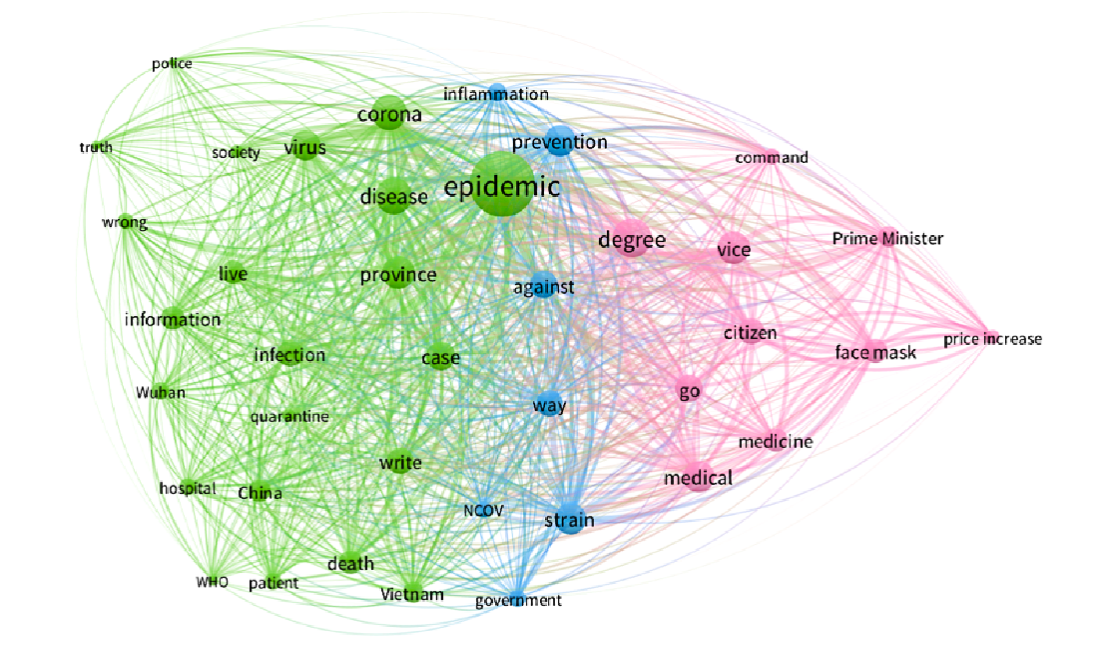
Co-occurrences of the top keywords in period 2. WHO: World Health Organization.

In period 2 (Figure 3), the most prominent keyword is “epidemic,” followed by the words “corona,” “disease,” “degree,” “prevention,” and “virus,” which reflect the public’s anxious reaction and the government’s quick policy response when the first two cases were found in Vietnam. The green cluster illustrates epidemic situation updates in China and the first 2 cases in Vietnam with keywords like “epidemic,” “corona,” “virus,” “infection,” “disease,” “province,” and “information.” The blue cluster represents the government’s epidemic prevention plan through the keywords “inflammation,” “government,” “against,” “prevention,” and “NCOV.” The pink cluster reflects when the prime minister issued a directive to sanction drugstores, which increased mask prices, as reflected in the keywords “vice,” “prime minister,” “command,” “face mask,” “price increase,” etc.

**Figure 4:**
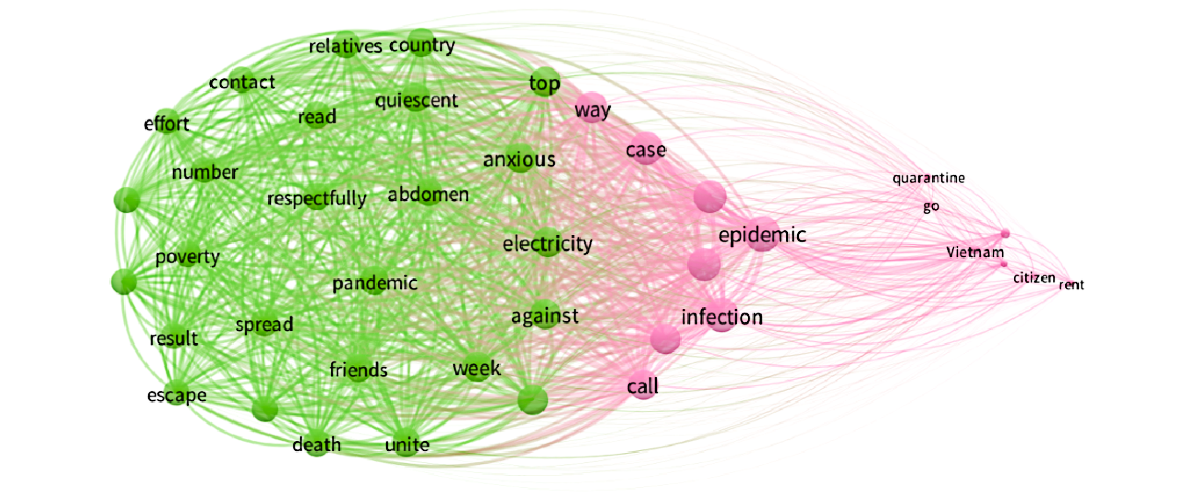
Co-occurrences of the top keywords in period 3.

Period 3 (Figure 4) was a short period prior to the pandemic’s peak in Vietnam during which no new cases were found. During this period, the keywords had almost the same weight. The most prominent keywords were still “epidemic,” followed by “starveling,” “infection,” “do not,” “case,” etc. The green cluster shows the respect for frontline workers’ efforts and calls for national spirit and unity to fight the pandemic via keywords including “regroup,” “respectfully,” “effort,” “quiescent,” “country,” “history,” “beaten,” etc. The pink cluster represents people’s cooperation with the government’s pandemic policy reaction, in particular quarantine. People called on each other to actively coordinate to isolate and prevent the epidemic from spreading more widely, including keywords “epidemic,” “do not,” “starveling,” “quarantine,” “case,” etc.

**Figure 5:**
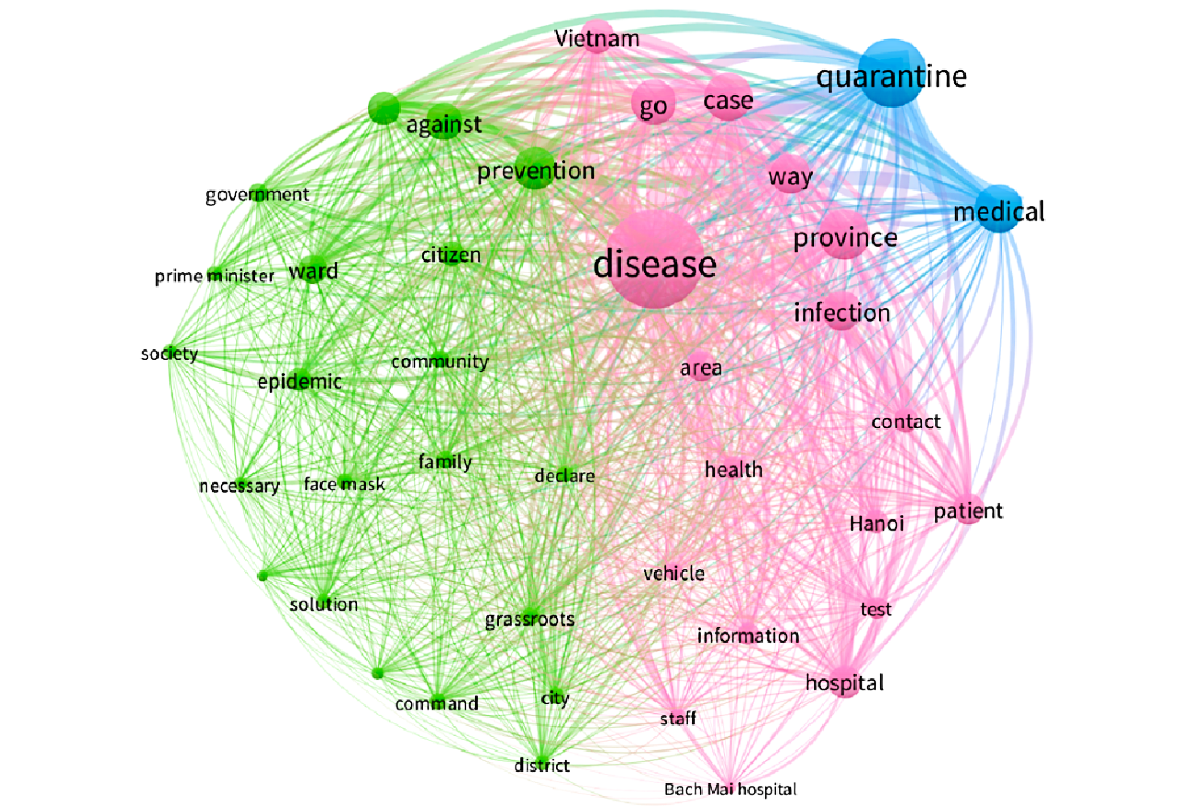
Co-occurrences of the top keywords in period 4.

Period 4 (Figure 5) was the peak of the pandemic in Vietnam after confirmation of the 17th infection case, with more reported afterward; the most prominent keyword was “disease,” followed by words like “quarantine,” “medical,” “province,” “case,” and “prevention.” To prevent the spread of the pandemic, Vietnam’s government provided solutions for pandemic prevention such as quarantine, temporary suspension of visas for all citizens of other countries who wanted to enter Vietnam, except for special cases. The government also introduced a health declaration app for those wishing to enter Vietnam at that time, along with the NCOVI app, which offers the public a reliable channel for information about COVID-19 and helps the government expedite contact tracing. The green cluster represents the *policy reaction* aspect, as expressed with keywords like “government,” “prime minister,” “command,” “prevention,” “face mask,” “against,” and “solution.” The pink cluster contained keywords related to the epicenter of the pandemic at Bach Mai hospital in Hanoi, such as “disease,” “Bach Mai hospital,” “Hanoi,” “case,” “infection,” “patient,” “staff,” “test,” and “contact.” The blue cluster represents the challenge of medical isolation through two keywords: “quarantine” and “medical.”

**Figure 6:**
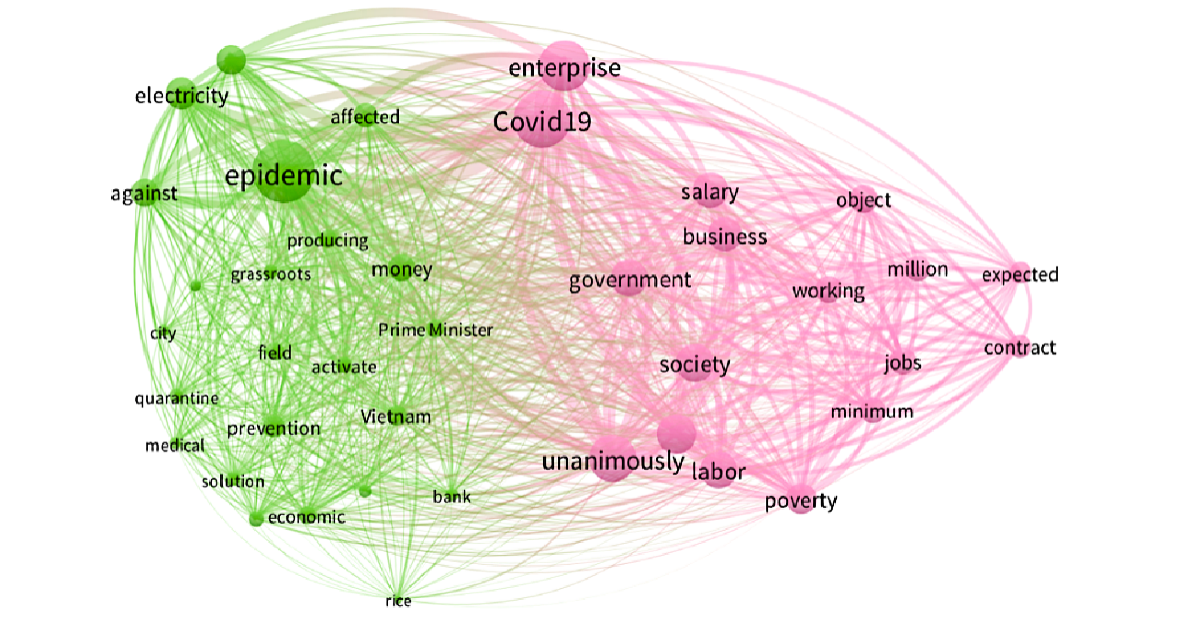
Co-occurrences of the top keywords in period 5.

During period 5 (Figure 6), the social isolation stage, the most prominent keywords were “epidemic,” “COVID-19,” “enterprise,” “unanimously,” etc. The green cluster represents the policy reaction aspect, in particular medical issues and economic solutions, such as supporting businesses and people working in production and consumption. Representative keywords included “prime minister,” “economic,” “solution,” “bank,” “electricity,” “rice,” etc. The pink cluster represents the economic concerns, as expressed by keywords including “enterprise,” “salary,” “business,” “labor,” “working,” “jobs,” “contract,” “society,” “poverty,” etc.

**Figure 7:**
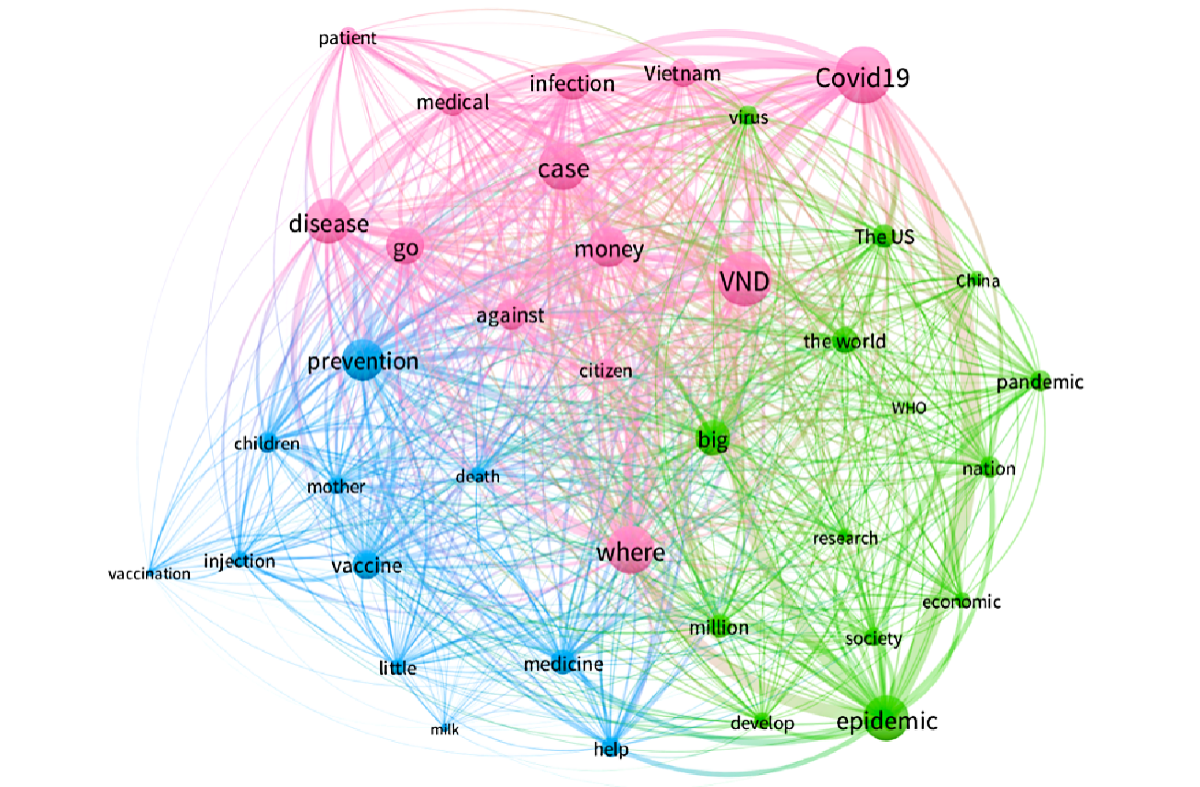
Co-occurrences of the top keywords in period 6. WHO: World Health Organization. VND: Vietnamese Dong.

The most prominent keywords in period 6 (Figure 7) included “COVID-19,” “disease,” “epidemic,” “case,” “prevention,” etc. This was the period when Vietnam controlled the epidemic well, with the result that there were no cases of community spread infection. People began to pay more attention to the challenge of COVID-19 vaccine research and development in Vietnam, which coincided with the period of vaccination against common diseases in young children, which was reflected in online public discussions. In addition, the public also paid more attention to pandemic prevention developments in Vietnam and the pandemic situation worldwide. The green cluster represents the global disease situation with keywords like “the US,” “China,” “epidemic,” “pandemic,” “WHO,” “research,” and “economic.” The pink cluster shows continued interest in COVID-19 cases and defense against disease in Vietnam via keywords such as “COVID-19,” “disease,” “go,” “where,” “infection,” “medical,” and “against.” The blue cluster represents concerns about vaccinating children against common diseases during the COVID-19 pandemic through keywords such as “vaccination,” “injection,” “children,” “prevention,” “death,” “mother,” and “help.”

**Figure 8:**
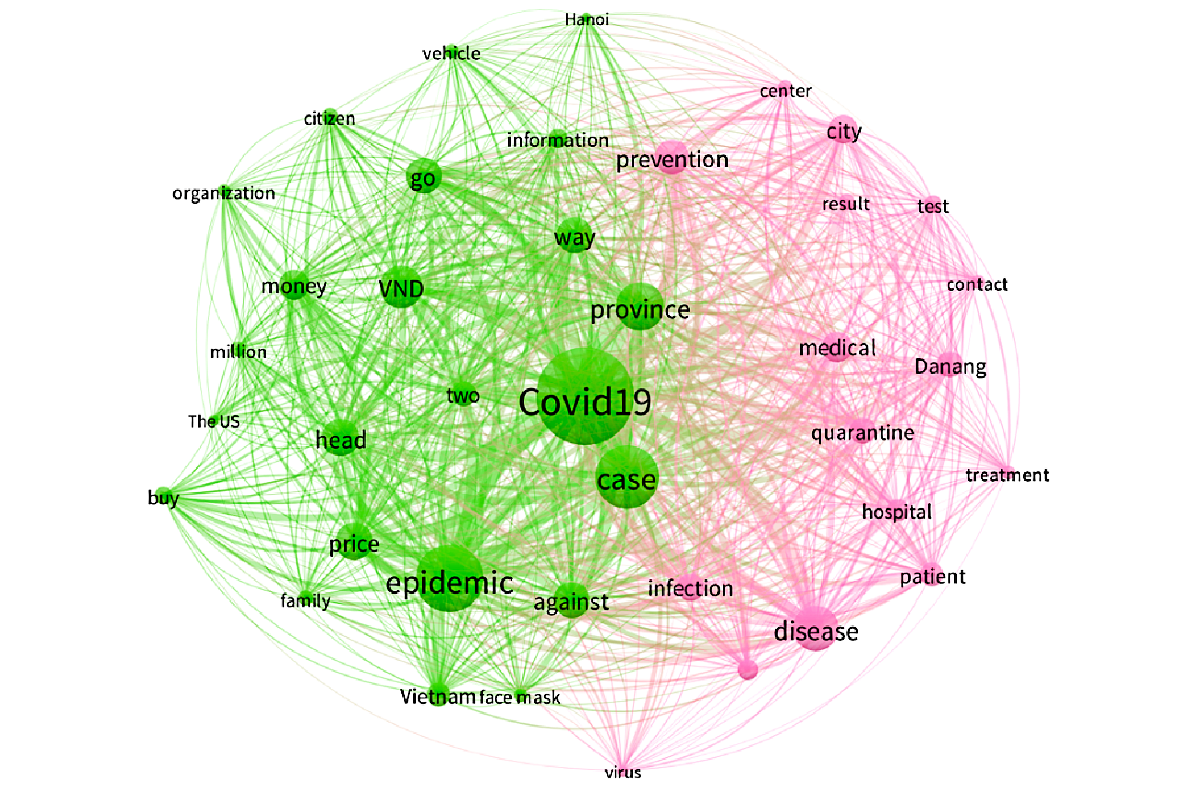
Co-occurrences of the top keywords in period 7.

The most prominent keyword in Vietnam during period 7 (Figure 8) was “COVID-19,” followed by words like “epidemic,” “case,” “province,” and “disease.” This period was marked by the re-emergence of cases in the community, so the public was most interested in two major topics. The first was the situation surrounding the Danang outbreak, which created the second pandemic wave in Vietnam (pink cluster). Keywords illustrating this event included “Danang,” “city,” “hospital,” “infection,” “test,” “quarantine,” etc. The second was concern about common issues related to COVID-19, such as raising the price of masks and information about preventing community disease spread (eg, finding people who would share a bus ride with patients who were infected; green cluster). Keywords included “Covid-19,” “price,” “face mask,” “Vietnam,” “against,” “epidemic,” “vehicle,” “go,” and “Hanoi.”

## Discussion

### Main Findings

The COVID-19 pandemic is a sensitive time, and the need for reliable sources to avoid an *infodemic* is understandable. Analyzing the public’s responses to the pandemic through cyber discussions can provide an overview of the pandemic’s impact on the public. In this study, we found four main conclusions that answered six research questions. First, based on the changing discussion trends gathered on the subject of COVID-19, 7 periods were determined based on events that can be further aggregated into two pandemic waves in Vietnam. Second, people engaged most with community pages on Facebook. However, the sources with the highest average interaction efficiency per article were government sources. Third, people’s attitudes when discussing the pandemic shifted from expressing negative to positive emotions (expression of neutral emotions remained stable). Fourth, the type of content that attracts the most interaction from people varies from time to time. Beyond that, we found that the issue-attention cycle occurred four times during the two COVID-19 pandemic waves in Vietnam. In each COVID-19 wave, the issue-attention cycle occurred twice, with a small cycle first, followed by a big cycle later.

Listening to people’s attitudes during a pandemic as expressed through their interactions on the internet can help governments and related agencies quickly adjust communication plans to lead people through the pandemic with better precision. This study provides valuable information to those concerned about the COVID-19 pandemic in general and the public’s response to an entirely new crisis in particular. Based on the results of this study, governments could use it as a reference to evaluate the efficiency of using big data to address public health management issues. This resource not only can be used as a reference to deal with future epidemic crises but also is a valuable comparison of public reaction toward the pandemic across countries.

#### Public Discussions on the Topic of COVID-19 on Social Media

The volume of public attention during the COVID-19 pandemic was substantial, with a total of 37,917,631 public mentions and 22,652,638 public posts during the research observation period from December 1, 2019, to November 13, 2020. During the peak period, we recorded more than 1,255,175 publicly discussed mentions showing particular interest in the pandemic; these mentions also demonstrated that the amount of pandemic-related information generated by the public is substantial. This can inadvertently create an information matrix or *infodemic* for people who feel confident in their abilities to search for information on the internet and who tend to trust the opinions of others.

#### Issue-Attention Cycles Occurring During the COVID-19 Pandemic

During the two COVID-19 pandemic waves in Vietnam from December 2019 to November 2020, the pattern of public attention looks similar to the issue-attention cycle described by Downs [23]. However, instead of only 1 cycle per pandemic wave, each pandemic wave had up to 2 cycles, with a smaller cycle occurring before the larger one. Moreover, a remarkable point is that the issue-attention cycles that occurred during the COVID-19 pandemic did not represent the main issue (the pandemic) but rather showed the subissues (real events) related to the main issue. Moreover, the last stage of each cycle was a transition between cycles. This means the last stage of this cycle may be the first stage of the cycle that occurs after it.

When the first COVID-19 cases were discovered in Wuhan, China, people were not too concerned about this strange disease, despite the attention given to it by the Vietnamese government, especially the Ministry of Health and related agencies. However, when Vietnam saw its first cases of infection, people began to pay more attention. Anxiety peaked when people became aware that this is a dangerous, contagious, potentially fatal disease and that there was no vaccine yet. The situation was eased when the government’s pandemic prevention responses were effective.

During the second COVID-19 pandemic wave in Vietnam, people remained interested in the pandemic but discussed it less on social networks. Public attention peaked with the first COVID-19–related deaths in Vietnam. Public attention then quickly dropped and diverted to other issues. This shows that although the second COVID-19 pandemic wave in Vietnam appeared to have a more negative factor (the first recorded deaths), the public’s attitude was not as intense as it had been during the first COVID-19 pandemic wave. This may be explained by people’s acceptance of the fact that death is a foreseeable outcome for patients infected with COVID-19 and at the same time an expression of *not feeling surprised* after 6 months living through the pandemic.

#### The Most Engaging Sources During the COVID-19 Pandemic

Per our data analysis, community pages on Facebook received the most total interaction from the public, likely because these aggregate information for the community with diverse content types. Each of these news sites usually post multiple articles per day on the same COVID-19 topic. However, in terms of average efficiency per article, government-controlled news sites outperformed other news sources. Drawing from this conclusion, we recommend that the government increase the number of articles posted to sources under its control to achieve the greatest dissemination of information to the community. In addition, the government can also coordinate with sources such as community pages and KOLs’ pages to quickly, accurately, and easily distribute disease information to the public.

Through our analysis, *the frames of communication* (top posts that gained the most interaction) can be used to explain public sentiment about the COVID-19 pandemic. We categorized COVID-19 topics garnering top public interest into 12 categories, based on the adoption of 7 types of COVID-19 information described by Li et al [46] and simultaneously developed 6 additional content type categories based on our data analysis processing. These categories included caution and advice; notifications or measures taken; donation of money, goods, or services; emotional support; help seeking; doubt casting and criticizing; counter rumors; policy reaction; international situation updates; medical issues, treatment, or vaccines; effects of the pandemic on the economy; and entertainment. When the pandemic first started in China, information about *cautions and advice* and *international situation updates* got the most attention. Negative emotions were just beginning to be expressed and did not prevail. However, negative emotions gradually increased when cases first appeared in Vietnam, and articles about *policy reaction* gained the most attention, followed by *doubt casting and criticizing* articles. During the peak of the first pandemic wave, negative emotions peaked as well, but the public still paid the most attention to *policy reactions* and the *emotional support* articles. The desire for negative emotions to subside was shown by the public giving the most attention to *emotional support* articles in addition to articles about *policy reaction* and *medical issues*. Although negative emotions persisted in the second wave of the pandemic in Vietnam, articles related to *entertainment* gained the most attention. This shows the public’s optimism during the crisis, as they have experienced the first wave of epidemics in the past and have hopes of a *new normal life* to come with the expectation of mass vaccine distribution next year.

### Limitations

This study has some limitations. First, despite using big data to analyze the phenomenon of public reaction toward the COVID-19 pandemic, some noise or spam remains in the data set; the SocialHeat tool could not completely filter these out due to technology limitations and the complexities of natural language. Though natural language has been applied and innovated daily in SocialHeat’s tool, some texts or paragraphs containing incorrect grammar, teen code, dialects, etc, could not be processed or categorized. It is also important to note that although the data set was pulled from diverse sources like Facebook, YouTube, news sites, etc, the observed format was text only. This means that other formats such as video with text or audio captions or images with textboxes were not analyzed by the SocialHeat tool. Hence, this led to a shortage in the final data set results such as sentiments categorized, extracted top sources, and extracted top posts.

Additionally, due to privacy policies, the data set can only collect data that is installed in public mode, especially for data obtained from social networking platforms like YouTube, Instagram, and Facebook. Moreover, because the data collection time is quite long (11 months), the amount of data poured into the system is large and requires a substantial amount of time for the system to process noise and spam, and give statistical results. This led to a situation in which we wanted to analyze the *top posts by mentions* in depth, but often encountered links that no longer worked because the owner of the post had changed the view mod from *public* to *friends* or *private*, or even deleted the post. This caused difficulties and data deficiencies in our analysis.

Finally, we have almost 38 million data in total, which the system could not process all at once due to technical limitations. Therefore, we could not extract top posts by mentions, top sources by mentions, or overall sentiment of all sources.

### Future Work

The topics discussed on the COVID-19 issue are varied. The classification of content groups as we propose in the study is still limited when it is impossible to analyze the public’s emotional index for each type of topic. Understanding the feelings of the community on specific topics related to the COVID-19 topic can help the government and stakeholders come up with precise and meticulous guidance on disease reactions. Therefore, we suggest that researchers focus on analyzing the public’s sentiment index for each type of topic that the public is discussing to come up with appropriate ideas and options to support the medical information management in pandemic times.

### Conclusions

Through our research, we found that using different types of information sources can be effective in different pandemic phases. The same goes for pandemic-related content types. We also highlighted hot spots of public concern regarding the COVID-19 pandemic. These results can help governments or health educators communicate pandemic prevention guidelines more effectively to the public. This is significant not only for prevention during the current COVID-19 pandemic but also could serve as a useful reference for the health crisis management field for potential diseases in the future.

#### Implications

Applying big data in infodemiology studies opens opportunities for getting better insights into a public reaction toward pandemics and related events. The government should take advantage of social platforms to effectively communicate health information, quickly address fake news, and give real-time response to the hot issues that the public needs to know during the pandemic. To achieve those goals, we suggest three key points to help government and stakeholders have better communication with the public during crisis events like the COVID-19 pandemic:

- Applying artificial intelligence tools in analyzing big data from social media platforms to collect public insights, determine appropriate cooperation channels in spreading news and guidelines, and effectively communicate about health information and instructions
- Promoting an official account of the Ministry of Health on different social media platforms to form the public’s habit of updating news from official sources, avoiding *infodemics* during the pandemic
- Collaborating with popular community and KOLs’ fan pages to spread information faster and wider to various reader segments

Big data is also meaningful for infodemiology studies. Applying big data allows researchers to have a wider view and easily compare the results across countries, regions, races, or cultures and lead to more research ideas such as descriptive studies or predicting public sentiments or public reactions about the pandemic.

## Appendix

Multimedia Appendix 1 Total mention trend line from December 1, 2019, to November 13, 2020.

## Abbreviations

API: application programming interface
KOL: key opinion leader
SARS: severe acute respiratory syndrome
WHO: World Health Organization
