# Supplementary figures and images for: Social Media Insights During the COVID-19 Pandemic: Infodemiology Study Using Big Data
Source: JMIR Med Inform. 2021 Jul 16;9(7):e27116. doi: 10.2196/27116 (PMC8288653; doi:10.2196/27116)

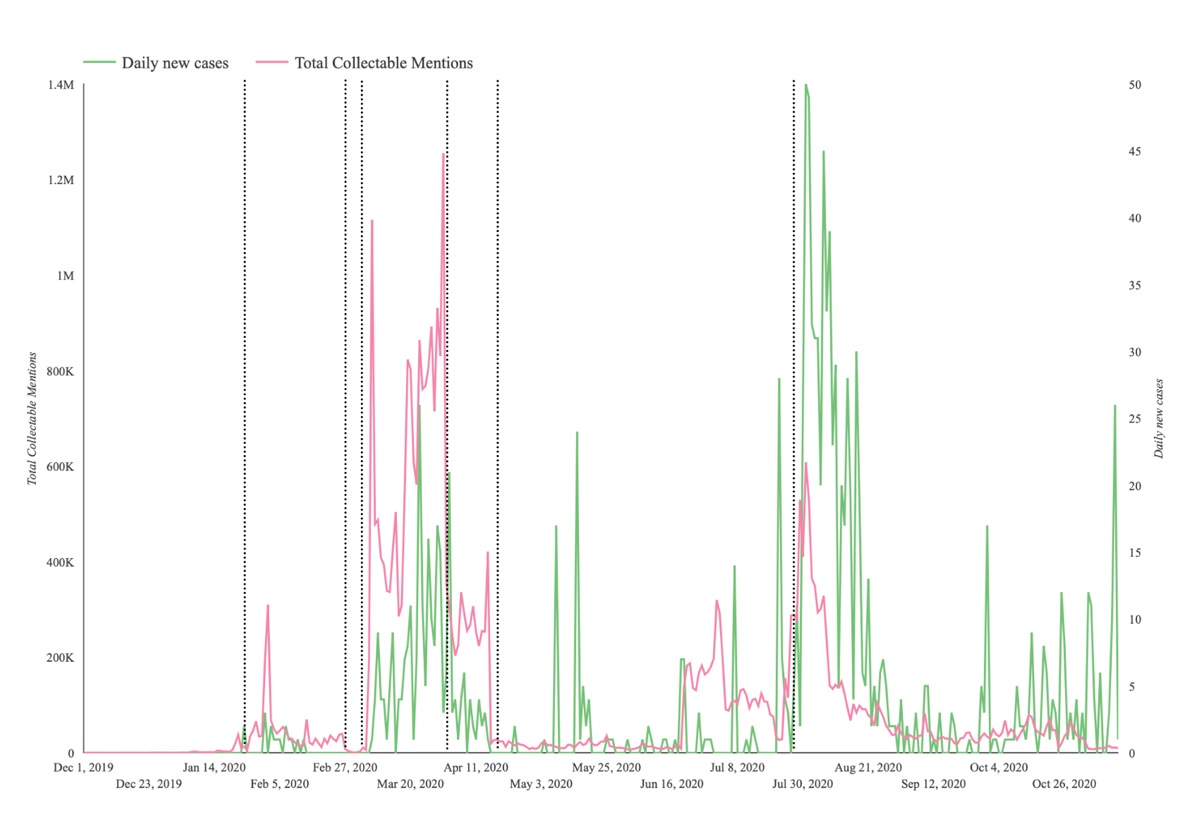

Supplement: Multimedia Appendix 1 [file medinform_v9i7e27116_app1.png]
